# Photocatalytic Oxidation of Carbon Monoxide Using Synergy of Redox-Separated Photocatalyst and Ozone

**DOI:** 10.3390/molecules27238482

**Published:** 2022-12-02

**Authors:** Seunghyun Weon

**Affiliations:** School of Health and Environmental Science, Korea University, Seoul 02841, Republic of Korea; s_weon@korea.ac.kr

**Keywords:** platinum, titanium dioxide, photocatalyst, carbon monoxide, indoor air

## Abstract

Separating the redox centers of photocatalysts is the most promising strategy to enhance photocatalytic oxidation efficiency. Herein, I investigate a site-selective loading of Pt on facet-engineered TiO_2_ to achieve carbon monoxide (CO) oxidation at room temperature. Spatially loaded Pt on {101} facets of TiO_2_ attracts photoinduced electrons efficiently. Thereby, oxygen dissociation is facilitated on the Pt surface, which is confirmed by enhanced oxidation of CO by 2.4 times compared to the benchmark of Pt/TiO_2_. The remaining holes on TiO_2_ can be utilized for the oxidation of various gaseous pollutants. Specifically, gaseous ozone, which is present in indoor and ambient air, is converted to a hydroxyl radical by reacting with the hole; thus, the poisoned Pt surface is continuously cleaned during the CO oxidation, as confirmed by in situ diffuse reflectance infrared transform spectroscopy. While randomly loaded Pt can act as recombination center, reducing photocatalytic activity, redox-separated photocatalyst enhances charge separation, boosting CO oxidation and catalyst regeneration via simultaneous ozone decomposition.

## 1. Introduction

Carbon monoxide (CO) in indoor air is regarded as silent killer that can cause both acute and chronic poisoning to human health. According to the Centers for Disease Control (CDC), approximately 50,000 people in the United States suffer from accidental CO poisoning, and more than 400 people die every year due to indoor CO pollution [1]. Since CO is colorless and odorless, it is hard for our senses to detect, and humans are prone to long-term exposure. Inhaled CO molecules displace oxygen molecules in the human body, leading to various respiratory disorders [2,3]. Worn or poorly tuned combustion devices (e.g., boilers, stoves, furnaces, etc.) that can cause incomplete burning are significant sources of indoor CO pollution. Due to the lack of functional groups of gaseous CO, air cleaners employing conventional filtration/adsorption technologies are ineffective at controlling indoor CO pollution [4,5]. Thus, the need for the development of selective CO-removal technology considering living space is attracting attention.

The oxidation of gas phase CO into CO_2_ is a thermodynamically favorable reaction; however, the high energy barrier of oxygen dissociation has to be overcome. Various supported noble metal catalysts such as Pt-, Pd-, and Au- have been developed to achieve CO elimination, but they still require an elevated temperature for oxygen dissociation, which is not suitable for indoor spaces [6,7,8]. Meanwhile, it has been reported that CO is oxidized through photocatalysis using noble-metal-supported photocatalysts [9,10,11]. For instance, Pt nanoparticles (Pt NPs) on the surface of TiO_2_ receive photoelectrons and provide catalytic sites to stabilize active oxygen species, thus enhancing the CO oxidation [12]. In contrast to the photocatalytic degradation of volatile organic compounds mediated through OH radicals [13,14], oxygen activation via photoelectrons is the main pathway for photocatalytic CO oxidation.

The separation of the redox sites of the photocatalyst has been a widely pursued strategy used to maximize the use of charge carriers [15]. Since photoelectrons are a main essential component of CO oxidation, Pt NPs decorated on the reduction sites of photocatalyst are recommended. Anatase TiO_2_ represents three major facets, including {101}, {010}, and {001}; these surface facets exhibit different surface energies of 0.44, 0.53, and 0.90 J/m^2^, respectively [16]. Due to bandgap differences, photoelectrons tend to transfer to {101} facets. In general, commercially available anatase TiO_2_ is randomly mixed with {101} and {001} facets, so the reduction and oxidation sites are not aligned. The facet-engineering of TiO_2_ with aligning {101} and {001} facets can successfully achieve selective loading of Pt at the reduction site. Electrons in the {101} facet surface tend to be trapped at a stable-surface Ti^3+^-bridging OH complex; reductive conversion reactions are easily facilitated on the {101} facet. Pt NPs tend to load on {101} facets, while {001} facets remain for oxidation. The separation of redox sites with the material facet-engineering allows efficient oxidation of CO and simultaneous gaseous pollutants.

In this study, I investigated a redox-separated photocatalyst to achieve CO oxidation at ambient temperature. The successful loading of Pt at the reduction site was achieved via facet-engineered TiO_2_ that combines reductive {101} facets and oxidative {001} facets. During the irradiation, Pt loaded of the reduction site was highly activated to dissociate oxygen molecules and thereby oxidize CO. Further, I explored a synergistic effect between CO oxidation and ozone (O_3_) removal, which are performed via photoelectrons and holes, respectively. Overall, a redox-separated photocatalyst can serve as a versatile platform for efficiently removing CO and ozone.

## 2. Result and Discussion

### 2.1. Structural Properties of Redox-Separated Photocatalysts

**Figure 1:**
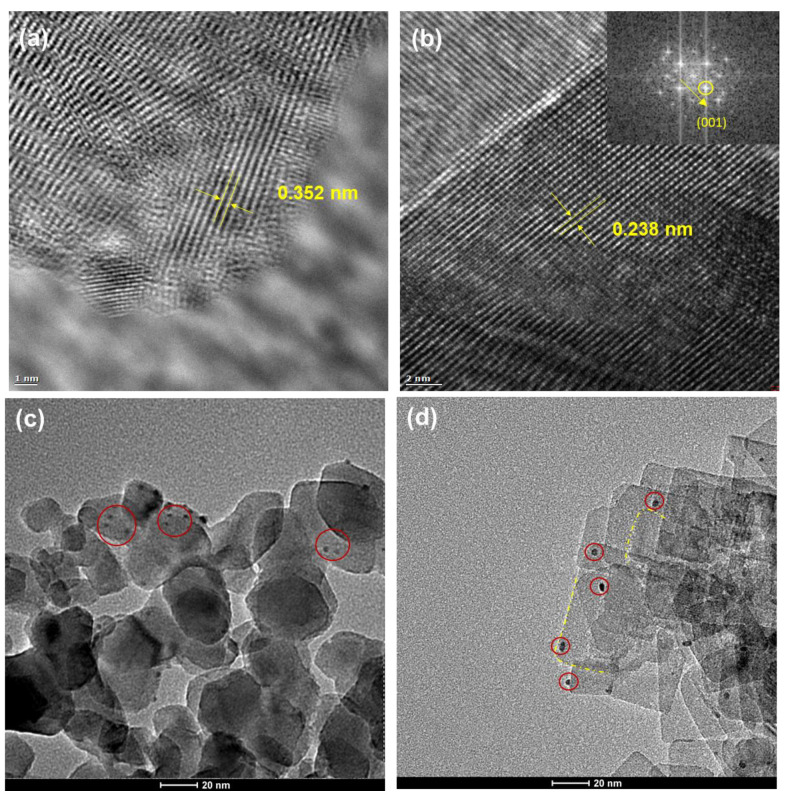
Magnified HR-TEM images obtained from (**a**) TiO_2_ and (**b**) facet-engineered TiO_2_. The inset of (**b**) shows the FFT (Fast Fourier Transform) pattern of facet-engineered TiO_2_. HR-TEM images obtained from (**c**) Pt/TiO_2_ and (**d**) Pt/facet-engineered TiO_2_. The concentration of Pt of each photocatalyst was fixed at 1.0 wt% with respect to TiO_2_ on a weight basis. Red circles represent Pt nanoparticles loaded onto TiO_2_. Yellow lines represent the edge of facet-engineered TiO_2_.

Facet-engineered TiO_2_ has a plate-like morphology with an average width of 48.30 nm and a thickness of 3.45 nm on the basis of obtained TEM images. The exposed surface of facet-engineered TiO_2_ is mainly composed of {001} facets, while the edge of facet-engineered TiO_2_ is mainly composed of {101} facets [17]. A magnified HR-TEM image of P25 shows that a lattice spacing of 0.352 nm corresponds to a (101) plane of anatase TiO_2_ (Figure 1a). In contrast, a magnified HR-TEM image of facet-engineered TiO_2_ shows a continuous lattice fringe with a spacing of 0.238 nm, corresponding to a (001) plane of anatase TiO_2_ (Figure 1b). This implies that the main plate of facet-engineered TiO_2_ is composed of {001} facets. Due to the complicated electron movements on the surface of TiO_2_, photodeposited Pt was positioned irregularly both in the center and on the edge of P25 (Figure 1c). The TEM image of Pt/Facet-engineered TiO_2_ clearly shows the presence of Pt NPs aligned on the edge of TiO_2_ (Figure 1d). Photoelectrons tend to move to edge of the facet-engineered TiO_2_; Pt deposits were selectively reduced in the edge positions. The average size of the Pt NPs of the Pt/TiO_2_ was estimated to be 2.7 ± 0.21 nm. As confirmed via ICP-OES, all 1.0 wt% Pt precursors used for photodeposition were loaded onto the surfaces of both the P25 and facet-engineered TiO_2_.

**Figure 2:**
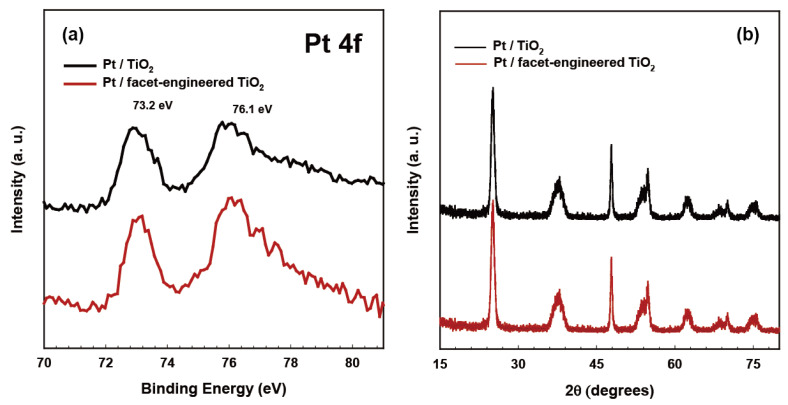
(**a**) XPS spectra and (**b**) XRD pattern of Pt 4f of Pt/TiO_2_ and Pt/facet-engineered TiO_2_.

To check the Pt’s oxidation state, XPS analyses on Pt 4f were conducted on both the Pt/TiO_2_ and Pt/facet-engineered TiO_2_ (Figure 2a). The XPS clearly showed binding energies at 73.2 eV and 76.1 eV of Pt [18,19,20]. There is no significant difference of XPS between Pt/TiO_2_ and Pt/facet-engineered TiO_2_, which implies oxidation states of Pt with the two photocatalysts are the same. Figure 2b shows the XRD patterns of Pt/TiO_2_ and Pt/facet-engineered TiO_2_. The XRD pattern of the Pt/facet-engineered TiO_2_ is the same as that of the Pt/TiO_2_, which implies that the photocatalyst structure did not collapsed during either the facet-engineering or the photodeposition. The structural properties of the Pt of the two synthesized photocatalysts were the same physiochemically, only the loading position was different.

### 2.2. Photocatalytic Oxidation of CO

**Figure 3:**
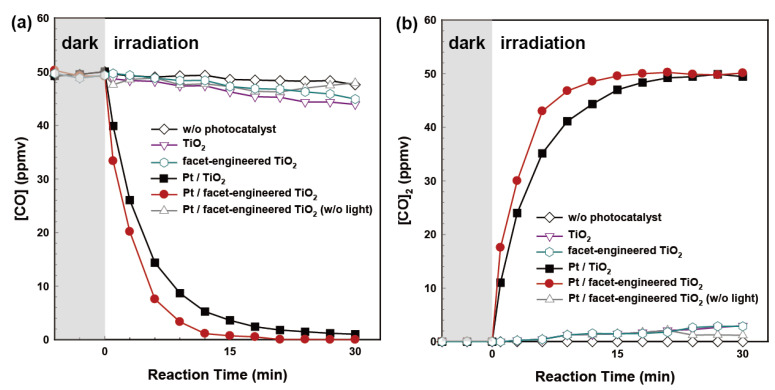
Time-dependent profiles of (**a**) photocatalytic oxidation of CO and (**b**) the concurrent production of CO_2_ on TiO_2_, facet-engineered TiO_2_, Pt/TiO_2_, Pt/facet-engineered TiO_2_, and control experiments (with/without photocatalyst film or irradiation). Experimental conditions: catalyst film size = 2 × 2 cm^2^, light source = UV LED (370 nm), and [CO]_0_ = 50 ppmv. Each oxidation experiment consisted of 10 min for adsorption equilibrium (dark) and 30 min for UV irradiation.

The TiO_2_, Pt/TiO_2_, and Pt/facet-engineered TiO_2_ films were tested for CO oxidation (Figure 3a). As a control experiment, CO oxidation was conducted without any photocatalytic films. During the 30 min of the reaction, the concentration of CO remained still. This implies CO is not oxidized by UV light irradiation without a catalytic material. The photocatalytic oxidation activities of TiO_2_ were negligible during the 30 min of the reaction, while that of Pt/TiO_2_ and Pt/facet-engineered TiO_2_ showed their superior activities for CO oxidation. The TiO_2_ with facet-engineering did not show any distinct CO oxidation. This implies Pt NPs are indispensable for CO oxidation to dissociate oxygen into active oxygen species [21]. The dissociative chemisorption of dioxygen is a prerequisite for CO oxidation, so Pt and photoelectrons are necessary for dissociation [22]. Among various noble metals such as Ag and Au, Pt-loaded TiO_2_ is known to exhibit superior photocatalytic oxidation efficiency for CO [10]. Without light irradiation, Pt/facet-engineered TiO_2_ was not activated, indicating that oxidation occurs only after photocatalytic activation. The concurrent CO_2_ generation was measured during CO oxidation (Figure 3b). The sum of the CO and CO_2_ concentrations over time was equal to the carbon inputs, meaning that the CO was converted directly to CO_2_ without any surface byproducts. To quantitatively compare the oxidation kinetics of CO using various photocatalysts, the pseudo first-order rate constants (for both Pt/TiO_2_ and Pt/facet-engineered TiO_2_) of CO oxidation were calculated. The pseudo first-order rate constant for CO oxidation increased approximately 2.4 times from 0.1326 ± 0.012 min^−1^ to 0.3212 ± 0.024 min^−1^ when using the Pt/facet-engineered TiO_2_. This implies that separating the reduction and oxidation sites significantly increases the photocatalytic CO oxidation efficiency.

### 2.3. Charge Transfer Properties of Redox-Separated Photocatalyst

**Figure 4:**
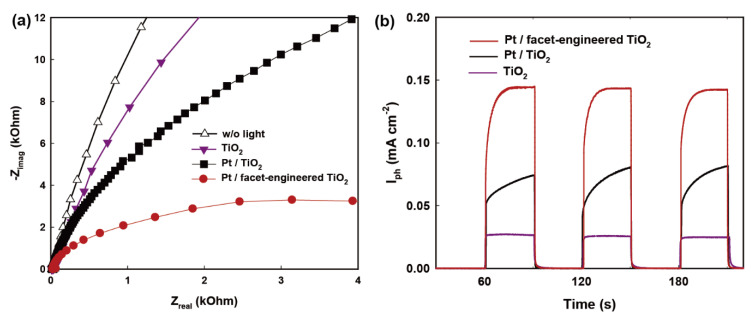
(**a**) Nyquist plots of prepared photocatalysts coated onto ITO from 1 MHz to 0.01 Hz at 0.5 V under UV irradiation. (**b**) Photocurrent production of prepared photocatalysts coated onto ITO under UV irradiation (60–90 s, 120–150 s, and 180–210 s).

Electrochemical analyses of prepared photocatalysts were carried out to unveil the role of Pt NPs according to the loading locations (random site vs. reduction site). EIS experiments were performed to analyze the charge transfer resistance between the catalyst surface and the electrolyte under UV irradiation (Figure 4a). The arc in the Nyquist plot appears due to interfacial charge transfer, so the diameter of the semicircle represents the quantitative amount of the photocatalysts’ resistance. The EIS results confirm that the resistance decreased with the introduction of Pt NPs and further decreased with facet-engineering. Interestingly, once Pt was deposited onto the reduction sites of the photocatalyst, the charge separation was maximized, and the interfacial resistance was reduced. The photocurrent was measured during intermittent UV irradiation of the synthesized photocatalysts to determine the number of available photoelectrons (Figure 4b). The Pt/facet-engineered TiO_2_ showed a significantly enhanced photocurrent compared to Pt/TiO_2_ and bare TiO_2_. The available photoelectrons were quantitatively enhanced 2.38 times compared to the randomly deposited case once Pt was deposited on the reduction site of TiO_2_. This trend is consistent with the CO oxidation experiment (Figure 3a), implying that the activation of Pt is preceded by photoelectrons in the photocatalytic CO oxidation reaction.

### 2.4. Ozone-Assisted Photocatalytic Oxidation of CO

**Figure 5:**
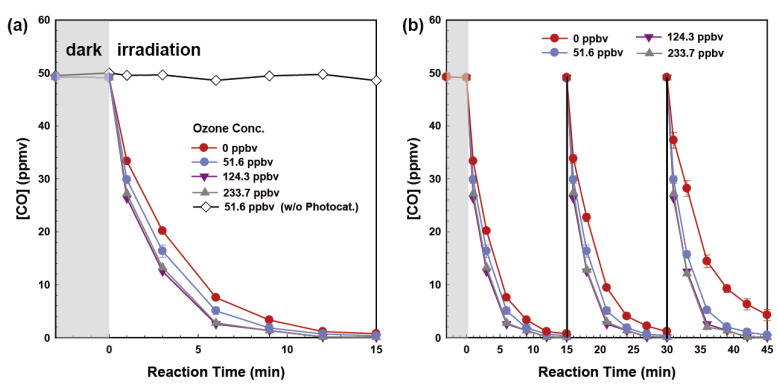
(**a**) Time-dependent profiles of photocatalytic oxidation of CO on Pt/facet-engineered TiO_2_ in several different initial ozone concentrations. (**b**) Repeated photocatalytic oxidation cycles of CO on Pt/facet-engineered TiO_2_ in several different initial ozone concentrations.

Ozone (O_3_) is another type of gaseous pollutant that humans can easily encounter indoors or in the atmosphere. O_3_ levels of several hundreds of ppbv are present indoors and outdoors and cause various chemical reactions [23]. When O_3_ is involved in photocatalytic reactions, the reaction mechanism is usually very complicated. O_3_ can be preferentially decomposed, preventing photocatalytic oxidation, or O_3_ can directly reduce air pollutants. To simulate the real-world conditions for CO oxidation, it is also necessary to determine the trend of oxidation reactions in the presence of small amounts of ozone. The CO oxidation was conducted using Pt/facet-engineered TiO_2_ with varying ozone concentrations (0, 51.6, 124.3, and 233.7 ppbv) (Figure 5a). The dark reaction between CO and O_2_ was negligible, even with the addition of O_3_. The ozone-enhanced CO photooxidation was observed for the Pt/facet-engineered TiO_2_. This implies that the electron and hole pairs generated by photon on the photocatalyst react with ozone directly or indirectly and then assist CO oxidation. Control experiments (with ozone and without a catalyst) showed no photocatalytic reaction both in dark and UV conditions. Therefore, ozonolysis of CO did not take a place, and a catalyst was essential for the ozone-enhanced effect. The ozone-induced enhancement in the photocatalytic reaction with CO was quickly saturated with an increasing concentration of ozone. The diffusion of ozone into the catalyst surface, up to 124 ppbv of ozone under UV irradiation, limited the photocatalytic rates. The role of O_3_ is to react with holes and facilitate the production of OH radicals and reactive oxygen species. These byproducts can directly participate in CO oxidation or can be utilized to remove surface deposits on a Pt surface. Repeated photocatalytic oxidation experiments (up to three cycles) were performed using Pt/facet-engineered TiO_2_ with varying ozone concentrations (Figure 5b).

**Figure 6:**
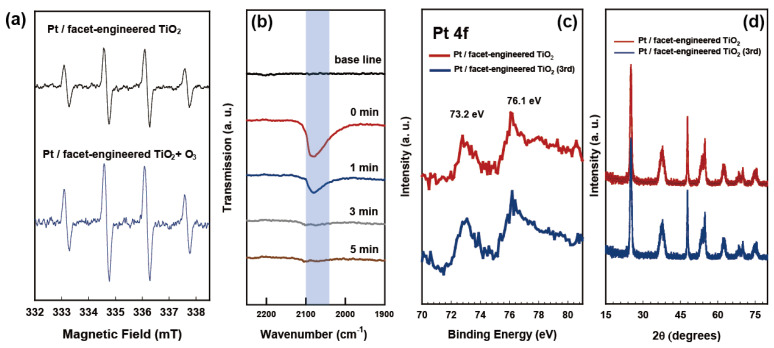
(**a**) Spin trap EPR of Pt/facet-engineered TiO_2_ with/without exposure to O_3_. Experimental conditions: light source = UV LED; illumination time: 15 min; catalyst dose = 0.25 g/L; [DMPO]_0_ = 30 mM; [O_3_] = 124.3 ppbv. (**b**) In situ Diffuse Reflectance Infrared Fourier Transform Spectroscopy DRIFTS (In situ DRIFTS) for Pt/facet-engineered TiO_2_ after three successive cycles of photocatalytic oxidation of CO. After exposing the sample to ozone flow with air for 1 min, 3 min, and 5 min. (**c**) XPS spectra and (**d**) XRD pattern of Pt 4f of Pt/facet-engineered TiO_2_, after 3rd photocatalysis cycle.

It is clear that the Pt/facet-engineered TiO_2_ showed a gradual decrease in successive cycles of CO oxidation. The XPS and XRD signals of the Pt/facet-engineered TiO_2_ after the three oxidation cycles did not show any changes, demonstrating the catalyst’s structural robustness (Figure 6c–d). The reduction occurred due to the fact that Pt tends to bind strongly with CO and deactivate oxygen dissociation [24]. Once O_3_ was introduced to the system, the photocatalytic CO oxidation efficiency was maintained during the cycles. This implies that O_3_ synergistically reacts with Pt/facet-engineered TiO_2_, which serves to enhance both the efficiency and durability of CO oxidation.

**Scheme 1:**
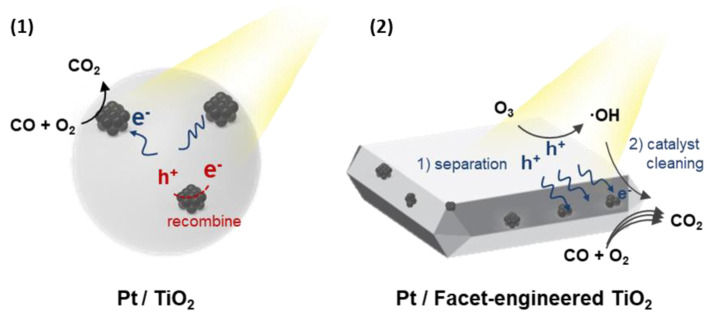
Schematic illustrations of (**1**) photocatalytic oxidation of CO using Pt/TiO_2_ and (**2**) site—selective removal of CO and O_3_ on Pt/facet-engineered TiO_2_.

To systematically unveil the role of O_3_ and Pt/facet-engineered TiO_2_, EPR and in situ DRIFTs were conducted on Pt/facet-engineered TiO_2_ with O_3_ exposure. EPR spin-trapping was used to verify the OH radical production of each catalyst system. After 15 min of irradiation, OH radicals were detected in the Pt/facet-engineered TiO_2_ (Figure 6a). Once O_3_ was introduced into the Pt/facet-engineered TiO_2_ along with the light irradiation, the OH radical signal was significantly enhanced. This implies that photogenerated holes can react with ozone and thereby produce more OH radicals. The in situ DRIFTS was taken using used Pt/facet-engineered TiO_2_ after three successive oxidation cycles which simulated poisoning with CO adsorption (Figure 6b). A broad peak containing linear CO on Pt NPs (2050–2080 cm^−1^) and Pt^δ+^-CO (2100 cm^−1^) was found [25]. After the ozone exposure, the broad peak gradually decreased over time, and the CO could be desorbed on the Pt surface. The poisoned Pt surface of Pt/facet-engineered TiO_2_ was regenerated upon the ozone exposure. The role of the Pt/facet-engineered TiO_2_ and O_3_ is schematically illustrated in Scheme 1. In general, Pt/TiO_2_ has mixed redox centers therefore, there are a lot of recombination of charge carriers, which significantly reduces photocatalytic activity. Facet-engineering with Pt loading can successfully divide reduction and oxidation centers. Therefore, CO can be efficiently oxidized at the Pt center that holds the photogenerated electrons. On the other hand, the remaining oxidation centers can react with various gaseous pollutants. In particular, O_3_ can react with a hole to produce an OH radical. The produced OH radical can sustainably regenerate the Pt poisoned surface, which is a major limitation for ambient catalytic CO oxidation. The material design that separates the redox center is a good strategy that shows synergy by simultaneously performing CO oxidation and O_3_ decomposition.

## 3. Experimental Section

### 3.1. Chemicals

All chemicals for material synthesis and analyses were used as received without any purification. Titanium butoxide (≥97.0%), ethanol (≥99.5%), sodium hydroxide (NaOH), hydrogen fluoride (HF), chloroplatinic acid hydrate (H_2_PtCl_6_), and methanol (HPLC grade, >99.9%) were purchased from Sigma Aldrich (St. Louis, MO, USA). Commercial TiO_2_ (P25) was purchased from Evonik (Essen, Germany). All aqueous solutions were prepared with ultrapure/deionized water (DI water, ≥18.2 MΩ·cm) from a Milli-Q system.

### 3.2. Preparation of Redox-Separated Photocatalysts

Facet-engineered TiO_2_ was prepared via a hydrothermal method [26]. A mixed solution of HF (3 mL) and titanium butoxide (25 mL) was poured into a 100 mL Teflon autoclave and heated for 24 h at 200 °C. The precipitate formed was separated using a centrifugation, washed with DI water, and then dried at oven (60 °C) for 12 h to yield a white powder. The loading of Pt on the reductive sites of the facet-engineered TiO_2_ was achieved by dispersing 0.1 g facet-engineered TiO_2_ with 320 μL H_2_PtCl_6_ (10 mM), which was added to the suspension. The mixture was stirred vigorously for 1 h at 1000 rpm. The suspension was subsequently irradiated with a 200 W mercury lamp for the photodeposition of Pt NP. The resulting material was collected via centrifugation, washed with DI water, and dried overnight at 60 °C to yield Pt/facet-engineered TiO_2_. As a comparison group, a commercial TiO_2_, P25, was used once for Pt deposition; this was named Pt/TiO_2_. For CO oxidation and electrochemical analyses, the photocatalysts were fixed and coated onto substrates. The synthesized catalysts were thoroughly mixed with ethanol to produce a homogeneous paste (0.15 g/mL), which was doctor-bladed onto a indium tin oxide (ITO) substrate of the size of 2 cm × 2 cm [27]. The resulting film used for the analyses were dried at 200 °C by flowing N_2_ for 2 h to remove residual ethanol.

### 3.3. CO Oxidation Measurements

The carbon monoxide (CO) oxidation was conducted at ambient conditions (room temperature and atmospheric pressure) in a closed-circulation reactor [27]. A Pyrex glass reactor (volume: 300 mL) with a quartz window (radius: 30 mm) was connected to photoacoustic gas monitor (INNOVA 1412i, LumaSense, Santa Clara, CA, USA) using a Teflon tube (radius: 2 mm). A magnetic bar was placed inside the reactor to circulate the air in it. A 370 nm-emitting UV-LED (ICN14D-096, Luna Fiber Optic Korea, Seul, Republic of Korea) was used as a light source to activate the photocatalysts. The intensity of the UV light flux at the surface of the photocatalyst was measured to be 16 mW/cm^2^ using a power meter (1815-C, Newport, Irvine, CA, USA). The photoacoustic gas monitor can measure the concentrations of CO, carbon dioxide (CO_2_), and water vapor simultaneously. The concentration of CO was adjusted by diluting standard gas (1000 ppmv CO in N_2_ as a carrier gas). The relative humidity was controlled atcirca 65 ± 10% by bubbling synthetic air through a stainless steel bottle containing DI water. O_3_ was introduced through the ozone generator (Evoqua) connected to the glass reactor. O_3_ was produced from the flow of oxygen standard gas (99.9%). The maximum production rate of O_3_ into the reactor was 1.6 g/min. The photocatalyst films of Pt/Facet-engineered TiO_2_, Pt/TiO_2_, and TiO_2_ were compared for CO oxidation under the same conditions. Before each experiment, the reactor was flushed with air to remove any remaining gases.

### 3.4. In Situ Diffuse Reflectance Infrared Fourier Transform Spectroscopy (In Situ DRIFTS)

In situ DRIFTS was performed using a FT-IR spectrometer (PerkinElmer, Waltham, MA, USA) equipped with a diffuse-reflectance cell (PIKE Technologies, Cottonwood, AZ, USA) with a ZnSe window. Powder catalysts were placed in the cell. CO of 100 ppmv was introduced into the cell by diluting the standard gas (300 ppmv CO in N_2_) with air. The relative humidity was adjusted by bubbling air through a stainless-steel bottle containing DI water. The in situ DRIFTS were collected after exposing the used sample (up to three successive CO oxidation cycles) to the flowing O_3_ stream to irradiation for 1 min, 3 min, and 5 min, respectively.

### 3.5. Material Characterizations

The high-resolution transmission electron microscopy (HR-TEM) images were taken using a JEM-2200FS (JEOL) operated at 200 kV, coupled with Cs correction. X-ray photoelectron spectroscopy (XPS) measurements were conducted using a Versa Probe Ⅱ Scanning XPS Microprobe (Physical Electronics (PHI)). X-ray diffraction (XRD) spectra were obtained using Cu-Kα radiation XRD (Mac Science Co., M18XHF, Tokyo, Japan). Electron paramagnetic resonance (EPR) measurements were analyzed using a JES-X310 spectrometer (JEOL, Tokyo, Japan). The Pt loading amount was measured using inductively coupled plasma optical emission spectrometry (ICP-OES-6300-Thermo Scientific, Waltham, MA, USA).

### 3.6. Photoelectrochemical Measurements

Photoelectrochemical (PEC) analyses were conducted using a potentiostat (Gamry, Warminster, PA, USA, Reference 600) connecting a three-electrode system. The prepared photocatalyst film, a Ag/AgCl electrode and Pt foil, were employed as a working, reference and a counter electrode, respectively. The electrochemical impedance spectroscopy (EIS) Nyquist plot was obtained using an alternating current (AC) voltage of 30 mV. PEC measurements were carried out with a potential of +0.5 V in the solution of 0.1 M NaClO_4_.

## 4. Conclusions

To boost the photocatalytic activity in material design, separating reduction and oxidation sites is an efficient method. Since the electronic structures and surface reactivity are different depending on crystal facets, facet-engineering can be used for separating oxidation and reduction sites [28]. Band alignment between different facets provides a route for charge carriers to separate. The {111} and {110} facets of rutile TiO_2_ are regarded as the oxidation and reduction facets, respectively [29,30]. As a major visible-light photocatalyst, four predominant exposed facets of BiVO_4_ and charge transfer properties have also been elucidated [31]. In addition, the simultaneous embedding of dual cocatalysts onto photocatalysts has been investigated with the aim of separating redox centers and enhancing photocatalytic activities [32]. Recently, oxidative and reductive cocatalysts were loaded onto 2D C_3_N_4_ to achieve facile water oxidation and H_2_O_2_ production. Cobalt single atoms were loaded onto oxidative sites for improving water oxidation efficiency, whereas anthraquinone was deposited onto reductive sites for enhancing photoreduction efficiency [15]. Here, I synthesized selectively loaded Pt on the reduction site of facet-engineered TiO_2_ and applied it to CO oxidation at room temperature. The CO was efficiently oxidized with enhanced oxygen dissociation, which occurs via photoelectrons transferred to Pt. Spatially separated Pt can efficiently draw electrons and utilize them for CO oxidation. The remaining holes can be utilized for ozone decomposition. Ozone was decomposed into OH radical, which can reactivate a Pt surface poisoned with CO. In general, CO oxidation using nanomaterials has a property whereby the efficiency gradually decreases under ambient conditions. This is because CO has a high affinity for Pt and covers the surface. The redox-separated photocatalyst capable of maximizing charge separation efficiency can oxidize CO while decomposing O_3_ via oxidative pathways. The Pt/facet-engineered TiO_2_ can serve as versatile platform that synergizes to oxidize CO and decompose O_3_, simultaneously.

## Data Availability

Not applicable.
